# Cost-effectiveness analysis of nonoperative management versus open and laparoscopic surgery for uncomplicated acute appendicitis in Colombia

**DOI:** 10.1186/s12962-021-00288-2

**Published:** 2021-06-10

**Authors:** César Augusto Guevara-Cuellar, María Paula Rengifo-Mosquera, Elizabeth Parody-Rúa

**Affiliations:** Faculty of Health Sciences, Universidad Icesi, Calle 18 No. 122-135 Pance, 70000 Cali, Colombia

**Keywords:** Cost-effectiveness, Nonoperative management, Open appendectomy, Laparoscopic appendectomy, Acute appendicitis

## Abstract

**Background:**

Traditionally, uncomplicated acute appendicitis (AA) has been treated with appendectomy. However, the surgical alternatives might carry out significant complications, impaired quality of life, and higher costs than nonoperative treatment. Consequently, it is necessary to evaluate the different therapeutic alternatives' cost-effectiveness in patients diagnosed with uncomplicated appendicitis.

**Methods:**

We performed a model-based cost-effectiveness analysis comparing nonoperative management (NOM) with open appendectomy (OA) and laparoscopic appendectomy (LA) in patients otherwise healthy adults aged 18–60 years with a diagnosis of uncomplicated AA from the payer´s perspective at the secondary and tertiary health care level. The time horizon was 5 years. A discount rate of 5% was applied to both costs and outcomes. The health outcomes were quality-adjusted life years (QALYs). Costs were identified, quantified, and valorized from a payer perspective; therefore, only direct health costs were included. An incremental analysis was estimated to determine the incremental cost-effectiveness ratio (ICER). In addition, the net monetary benefit (NMB) was calculated for each alternative using a willingness to pay lower than one gross domestic product. A deterministic and probabilistic sensitivity analysis was performed.

**Methods:**

We performed a model-based cost-effectiveness analysis comparing nonoperative management (NOM) with open appendectomy (OA) and laparoscopic appendectomy (LA) in patients otherwise healthy adults aged 18–60 years with a diagnosis of uncomplicated AA from the payer’s perspective at the secondary and tertiary health care level. The time horizon was five years. A discount rate of 5% was applied to both costs and outcomes. The health outcomes were quality-adjusted life years (QALYs). Costs were identified, quantified, and valorized from a payer perspective; therefore, only direct health costs were included. An incremental analysis was estimated to determine the incremental cost-effectiveness ratio (ICER). In addition, the net monetary benefit (NMB) was calculated for each alternative using a willingness to pay lower than one gross domestic product. A deterministic and probabilistic sensitivity analysis was performed.

**Results:**

LA presents a lower cost ($363 ± 35) than OA ($384 ± 41) and NOM ($392 ± 44). NOM exhibited higher QALYs (3.3332 ± 0.0276) in contrast with LA (3.3310 ± 0.057) and OA (3.3261 ± 0.0707). LA dominated the OA. The ICER between LA and NOM was $24,000/QALY. LA has a 52% probability of generating the highest NMB versus its counterparts, followed by NOM (30%) and OA (18%). There is a probability of 0.69 that laparoscopy generates more significant benefit than medical management. The mean value of that incremental NMB would be $93.7 per patient.

**Conclusions:**

LA is a cost-effectiveness alternative in the management of patients with uncomplicated AA. Besides, LA has a high probability of producing more significant monetary benefits than NOM and OA from the payer’s perspective in the Colombian health system.

**Supplementary Information:**

The online version contains supplementary material available at 10.1186/s12962-021-00288-2.

## Background

Acute appendicitis (AA) is one of the most common causes of abdominal pain. The European Association for Endoscopic Surgery estimates this condition affects from 5.7 to 57 per 100,000 people each year. The lifetime risk is about 16.3% in industrialized countries [1], 8.6% for men, and 6.7% for women in developing countries [2]. Furthermore, the total medical costs associated with this condition oscillate between $5989 and $6075 per patient in developing countries [3, 4]. In Colombia, it is estimated that the cumulative costs generated by open (OA) and laparoscopic appendectomy (LA) are US$65,753 and $66,425, respectively, in 2013. Finally, complications can generate cumulative costs of approximately $297 for OA and $271 for LA in that period [5]. Despite being a benign condition, its high incidence and risk of complications represent a significant financial burden for healthcare systems.

Traditionally, AA has been treated surgically. The surgical approach has been preferred because of the high success rate, eradication of the underlying cause, and the relatively low procedure's complexity [6]. Indeed, around 300,000 appendectomies are performed annually only in the United States, preferring the LA [7]. However, the technological restrictions, the cost of some supplies, and the lack of trained staff are barriers to LA's wider adoption [8, 9]. For this reason, a significant proportion of appendectomies are still performed through an open approach worldwide [5, 10]. An example of this is a recent study in Bogota, Colombia, where 65,625 subjects underwent appendectomy between 2013 and 2015. Of these, 92.9% underwent OA, and only 7.1% underwent LA [2]. Nevertheless, OA presents a higher risk of complications such as infection of the surgical site, incisional hernias, abdominal pain, and obstructive symptoms [11].

In addition to the above, some situations have questioned traditional surgical management and propose alternative therapeutic approaches. Firstly, there are concerns about the safety of surgical treatment in high-risk surgical patients. Secondly, recent studies suggest that uncomplicated AA may be a condition with a high likelihood of spontaneous remission with supportive care alone [12]. Finally, several well-designed controlled clinical studies have shown the potential benefits of nonoperative management (NOM). The most recent long-term Appendicitis Accuta Trial (APPAC) compared antibiotic versus surgical management. In this study, NOM presented a success rate of 72.7% within 5 years [13]. Additionally, several systematic reviews have reported clinical benefits of NOM versus surgical approach [1, 6, 14–21].

A review of the literature shows that NOM brings certain advantages over appendectomy. These advantages include a significantly lower overall complication rate in the short term, a reduction of approximately 50% or more in costs, and a lower disability than surgery [3]. Additionally, there are other variables in which no statistically significant difference has been found between the two alternatives, such as hospital stay length [11, 22]. However, NOM has disadvantages, such as a significant probability of evolution to complicated appendicitis and recurrence, leading to incurring cost overruns.

This dilemma requires evaluating the long-term impact and economic implications of NOM of uncomplicated AA. Although cost-effectiveness analyses for this condition have been carried out in developed and developing countries, almost all evaluated NOM versus LA only [3–5, 23–25]. OA's exclusion in these evaluations might not represent the daily clinical practice in many developing countries where this approach is routine [10]. In this sense, our study seeks to evaluate the three alternatives simultaneously.

This study's objective was to evaluate the cost-effectiveness of NOM compared to OA and LA in patients diagnosed with uncomplicated AA in the Colombian health system.

## Methods

### Statements

A model-based cost-effectiveness analysis was conducted for estimating the efficiency of OA, LA, and NOM in the treatment of uncomplicated AA from the perspective of the public payer in the Colombian Health System. The Colombian health system is based on a market mechanism regulated by the Ministry of Health. There are public and private entities responsible for the insurance and provision of health services. The primary sources of financing are the quotations of employees and employers, who finance the private contributory regime, and fiscal resources obtained through general taxes, which finance the public subsidized regime. We refer the reader to the corresponding literature for more details on the Colombian health care system [26, 27].

The national methodological guideline for economic evaluation was followed [28]. This guideline recommends QALYs to measure health outcomes, adopt a discount rate for costs and outcomes similar to that adopted by countries in the region (3–5%), and a cost-effectiveness threshold of less than one Gross Domestic Product (GDP) per capita.

### Design and structure of the model

A literature review was conducted to identify published model-based economic evaluations. Although four models were found, no one was employed in our economic evaluation because they evaluated complicated appendicitis, prophylactic appendectomy, and different length of hospital stay giving antibiotic therapy [4, 23, 29, 30]. For this reason, a de novo decision model was designed.

The model evaluated three therapeutic alternatives for uncomplicated AA. Two options are surgical (OA and LA), which include short-term postoperative complications. The criteria for defining the realization of OA or LA are those reported in the POSAW study [6, 10]. OA must be performed in those hospitals where laparoscopic equipment or surgeons trained in laparoscopy are not available. Additionally, the surgeon may decide to perform OA because of the perception that this alternative has shorter operative and anesthetic times or lower risk of intra-abdominal abscess [13, 31]. Short-term postoperative complications included in the model were operative site infection, intra-abdominal abscess,and ileus [13]. Long-term complications (e.g., incisional hernias or fistulas) and death were not included in the model because of their low probability of occurrence in patients with uncomplicated AA [13, 31].

**Fig. 1:**
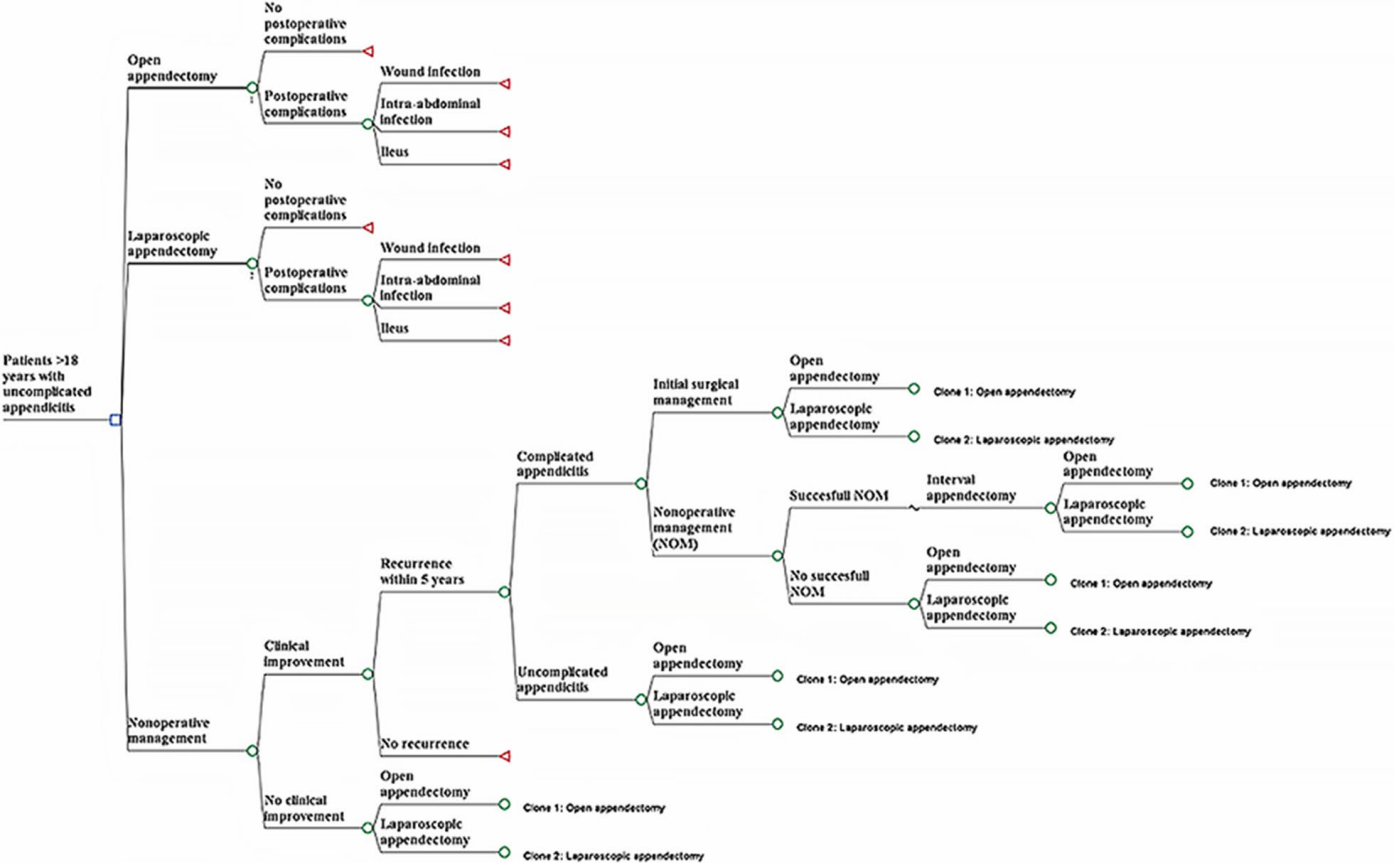
Structure of the model

The third alternative is NOM, which aims to reduce inflammation with the administration of antibiotics and other non-surgical interventions (e.g., analgesics, intravenous fluids). The absence of clinical improvement or absence of improvement in the diagnostic aids leads to realizing an appendectomy. The clinical improvement allows the patient to remain with the appendix. However, this permanence generates a risk of long-term recurrences or develops to complicated AA (i.e., presence of phlegmon, abscess or mass). For this reason, the model also included medical management (i.e. antibiotics plus percutaneous drainage) followed by the interval appendectomy in this case. However, the initial surgical management without medical treatment of complicated appendicitis also was considered. The inclusion of the two alternatives for managing complicated AA is because there is controversy about performing interval appendectomy in patients with complicated AA [32–34]. A decision tree was considered appropriate for modeling because events in uncomplicated AA usually have a short duration and rapid resolution. Figure 1 displays the model structure.

The model's assumptions were as follows: (I) The management of intra-abdominal abscess was the same regardless of the type of surgery. (II) Death was not included because it has a very low probability (< 1%) [13]. (III) The incisional hernia and the enteric fistula were not considered because they are negligible for both surgical procedures (0.4% and 0.5%) [35]. (IV) Abdominal sepsis was not included because of its very low incidence in uncomplicated AA [36]. (V) Postoperative ileus is managed within the same hospitalization without considerably affecting the quality of life.

The model was tested for face and external validation. For face validity, the structure of the model was designed based on updated clinical practice guidelines and protocols. Subsequently, the structure was repeatedly reviewed with a surgeon who approved the different courses of action of the tree. These courses of action had to represent the usual clinical practice in Colombia. For external validation, critical parameters of the model were selected: incidence of complications for OA and LA; recurrence of appendicitis at five years in patients who underwent NOM, and probability of complicated appendicitis in case of recurrence. The 95% confidence interval was calculated for each parameter. Calibration was not performed if there was overlap between the model and study confidence intervals. The results of the external validation are shown in Additional file 1: Table S1.

### Population, setting, subgroups and alternatives

The hypothetic population evaluated by our model included otherwise healthy adults aged 18 years to 60 years with a diagnosis of uncomplicated AA. Uncomplicated AA was defined as the absence of clinical signs suggestive of peritonitis and no evidence of inflammatory mass, phlegmon, abscess, or appendicolith in abdominal imaging. A subgroup analysis in older patients was not conducted because of a lack of long-term clinical data for patients older than 60 years. The context of the evaluation is the secondary and tertiary levels of the public health care system.

The alternatives included surgical and medical options. The surgical procedures evaluated were OA and LA. The NOM includes clinical observation in the hospital for at least 72 h, assessment by a surgeon, routine diagnostic aids, and antibiotics administration. The antibiotics regimen evaluated included ertapenem (1gr c/24 h × 3 days), levofloxacin (500 mg c/24 h × 7 days) + metronidazole (500 mg c/8 h × 7 days). These alternatives were extracted from clinical literature [11]. Besides, expert surgeons validated that those represent the routine clinical practice in our context. Other antibiotic regimens were not evaluated due to the absence of long-term clinical data.

### Model inputs

Costs were identified, quantified, and valorized from a payer perspective; therefore, only direct health costs were included.

Direct costs are valued by tariffs regulated by the Ministry of Health and are considered acceptable proxies for the production costs of health services. The most commonly used tariff for valuing health services (i.e., diagnostic and therapeutical procedures) is the *Manual Tarifario ISS* [37]. Some health services are valued as a package of services and others on a per-event basis. The payer and the provider agree on a value for a predetermined set of services in a package. Services not included must be valued and paid for additionally. In pay-per-event, each service is priced independently, and the total cost is the sum of the individual costs of each service. The unitary costs for medications were obtained from the *Sistema de Informacion de Medicamentos* [38]*.*

According to the tariff, OA and LA are valued as a package. The package for OA includes pre-surgical evaluation by the surgeon and anesthesiologist, performance of the procedure, post-surgical controls, operating room fees, surgical supplies (e.g., gauze, compresses, disposable clothing), and post-surgical hospitalization days. Post-surgical complications are not included in the package.

The value of the package for LA is the same value for OA, but the value of the single disposable trocar is added.

The NOM and complications are not valued as a package but as an event. For this reason, each resource used is valued independently, and the total cost will depend on the number of resources consumed. The identification and quantification of resources were obtained from the clinical practice guidelines and from an expert who validated them [6].

The resources quantified and valued for NOM included hospitalization, evaluation by a surgeon, fluids, analgesics, diagnostic aids (C-reactive protein, hemogram), and antibiotics (i.e., ertapenem, levofloxacin, and metronidazole).

In wound infection, antibiotics (i.e., cephalexin), medical evaluation, and analgesic were valued. For intra-abdominal infection and complicated AA, the resources valued were hospitalization, percutaneous drainage guided by ultrasound, antibiotics (i.e., ceftriaxone plus metronidazole), diagnostic aids, and evaluation by a surgeon. Finally, the resources valued in ileus were hospitalization, fluids, analgesics, diagnostic aids, and nasogastric tube placement.

Total cost was calculated by multiplying the unit cost by the units required of each resource. The costs were expressed US dollars at the current exchange rate in January 2021, reported by the Central Bank of Colombia (1 USD = COP 3478) [39]. Following Colombia's methodological guide for economic evaluations, all costs were adjusted by 30% for inflation [28].

The resources used in each alternative are shown in Additional file 2: Table S2.

The health outcomes were quality-adjusted life years (QALYs) according to the methodological guidelines. Given the absence of utility and disutility data for the health states in our country, the utility weights were extracted from the Cost-Effectiveness Analysis Registry of the Tufts Medical Center [40]. The QALYs were obtained by multiplying each event/state's utility by the length of time expressed in years.

The utilities used in each event are shown in Additional file 3: Table S3.

The time horizon was five years. This horizon was chosen because some late complications can occur during this period in patients managed with NOM [13]. A discount rate of 5% was applied to both costs and outcomes. This rate is recommended for the methodological guidelines for economic evaluation in Colombia [28].

The probabilities of each of the complications for OA were obtained from systematic reviews [14–16, 18, 20, 21, 36, 41–43]. The probabilities of LA complications were calculated by multiplying the probability of OA complication by the relative risk obtained from meta-analyses [14–16, 18, 20, 21, 36, 41–43]. The probabilities of clinical improvement, recurrence, and complicated appendicitis with NOM were obtained from the Salminen et al. [13]. The probabilities of selecting open or laparoscopic appendectomy or medical or surgical management of complicated appendicitis were obtained from observational studies [10, 31, 44–47].

### Incremental analysis

An incremental analysis was estimated to determine the incremental cost-effectiveness ratio (ICER).

This ratio is expressed mathematically in the following expression:

$$ICER=\frac{Incremental \, cost}{Incremental \, QALYs}=\frac{{Cost}_{B}-{Cost}_{A}}{{QALY}_{B}-{QALY}_{A}}$$

In addition, the net monetary benefit (NMB) was calculated for each alternative. This benefit is mathematically expressed as follows:

$$NMB=\Delta E\lambda -\Delta C$$

ΔE indicates the incremental effect (i.e., the difference of QALYs between two alternatives), ΔC indicates the incremental cost, and λ means the willingness to pay. The York Health Economic Consortium defines the NMB as "…a summary statistic that represents the value of an intervention in monetary terms when a willingness to pay threshold for a unit of benefit is known." [48].

The willingness to pay threshold was $6667. This threshold is equivalent to 1 GDP per capita in Colombia. The Colombian methodological guide for economic evaluations established this threshold to consider a technology highly cost-effective following the recommendations of the World Health Organization [28, 49, 50].

### Uncertainty analysis

A deterministic and probabilistic sensitivity analysis was performed. The deterministic analysis was a one-way sensitivity analysis whose objective was to identify which input affects the ICER. The results were graphed in a tornado plot for the essential variables. If the input is modifiable, a threshold analysis was performed to identify the value at which a non-cost effective alternative could become cost-effective.

The stochastic and parameter uncertainties were evaluated through the first- and second-order Monte Carlo simulations. A first-order microsimulation was performed with 1000 trials for each alternative to evaluate stochastic uncertainty (i.e., random variability in outcomes between identical patients). For this purpose, random utility values were assigned to each trial when entering the model. The parameter uncertainty was evaluated with a second-order Monte Carlo simulation with 10,000 iterations. A beta-distribution represents the uncertainty in utility and probability because these are binomial parameters constricted in the interval from zero to one. A Dirichlet distribution, a multivariant generalization of beta distribution, was used to model polytomous events (e.g., short-term complications in surgical alternatives). The Poisson distribution was used to model the number of resources. Resource values were modeled using a uniform distribution. Unfortunately, the resource values in the national tariffs are reported as a single value, with no mean or standard deviation to support the use of gamma or log-normal distribution to model this parameter. The relative risks were modeled using a uniform distribution, where lower and upper values correspond to the 95% confidence interval reported in the meta-analysis. The distribution parameters are presented in Additional file 4: Table S4. Finally, a simulation with 100,000 trials was performed to evaluate the effects on incremental cost in a hypothetical population with this number of patients.

The model was evaluated with TreeAge Health Pro 2020 software (TreeAge Software, Inc., USA).

This study did not require ethical approval because there was no participation of humans or animals.

## Results

The deterministic incremental analysis is presented in Table 1. This analysis revealed that LA dominates OA. Contrastingly, the NOM produced 0.003 more QALYs per patient (discounted) than LA but at a higher discounted incremental cost ($61 per patient). The ICER of $24,000/QALY additional is above the willingness to pay 1GDP per capita in Colombia ($6667/QALY additional). Therefore, NOM would not be considered an efficient alternative for that willingness to pay.

**Table 1:** Incremental analysis of the alternatives in treatment for uncomplicated AA

Alternatives	Undiscounted					Discounted				
	Mean cost^a^	Incremental cost	Mean QALYs	Incremental QALYs,	ICER ($/QALY)	Mean cost	Incremental cost	Mean QALYs	Incremental QALYs,	ICER($/QALY)
Laparoscopic appendectomy	308		4.9863			308		4.022		
Open appendectomy	309	1	4.9795	− 96	Absolute dominance	309	1	4.0966	− 0.0056	Absolute dominance
Nonoperative management	415	106	4.9899	0.0104	10.192	369	60	4.1052	0.0085	7.059

	Undiscounted					Discounted				
	Mean cost	Incremental cost	Mean QALYs	Incremental QALYs,	ICER($/QALY)	Mean cost	Incremental cost	Mean QALYs	Incremental QALYs,	ICER($/QALY)
Laparoscopic appendectomy	308		4.9863			308		4.1022		
Nonoperative management	415	107	4.9899	0.0036	29.722	369	72	4.1052	0.003	24.000

*QALYs* quality-adjusted life-years, *ICER* incremental cost effectiveness ratio

^a^Cost: US dollars (1 US dollar = 3478 COP)

The deterministic sensitivity analysis revealed that NMB is influenced by the cost of LA, NOM, and hospitalization time in LA. It is observed that when the costs of LA range between $150 and 350, the NMB ranges between $27,153 and $26,910. Similarly, the analysis shows that a decrease in LA hospitalization time by two days could increase the NMB by only 0.05% (from $27,041 to $27,055). Finally, the reduction of medical management costs by $200 produces an increase in NMB from $26,908 to $27,108. The tornado graph and the threshold analysis are available in the (Addditional file 5: Figures S1–S3). These results show that the reduction in costs of LA could generate the most significant variability in NMB.

Table 2 presents the results of the probabilistic sensitivity analysis. The Monte Carlo simulation reveals that LA presents a lower cost ($363 ± 35) than OA ($384 ± 41) and NOM ($392 ± 44). On the other hand, NOM exhibited higher QALYs (3.3332 ± 0.0276) in contrast with LA (3.3310 ± 0.057) and OA (3.3261 ± 0.0707). Histograms for each of these variables can be found in Addditional file 5: Figures S4–S20.

**Table 2:** Results of probabilistic sensitivity analysis

	Open appendectomy	Laparoscopic appendectomy	Nonoperative management
*Cost*^a^* (USD$)*			
Mean	384	363	392
Std Deviation	41	35	44
Minimum	295	295	264
Median	377	359	389
Maximum	573	359	635
*QALYs*			
Mean	3,3261	3,3310	3,3332
Std Deviation	0,0707	0,0570	0,0276
Minimum	2,3643	2,4987	2,8988
Median	3,3417	3,3417	3,3417
Maximum	3,3417	3,3417	3,3417

*QALYs* quality-adjusted life-years

^a^Cost: US dollars (1 US dollar = 3478 COP)

**Fig. 2:**
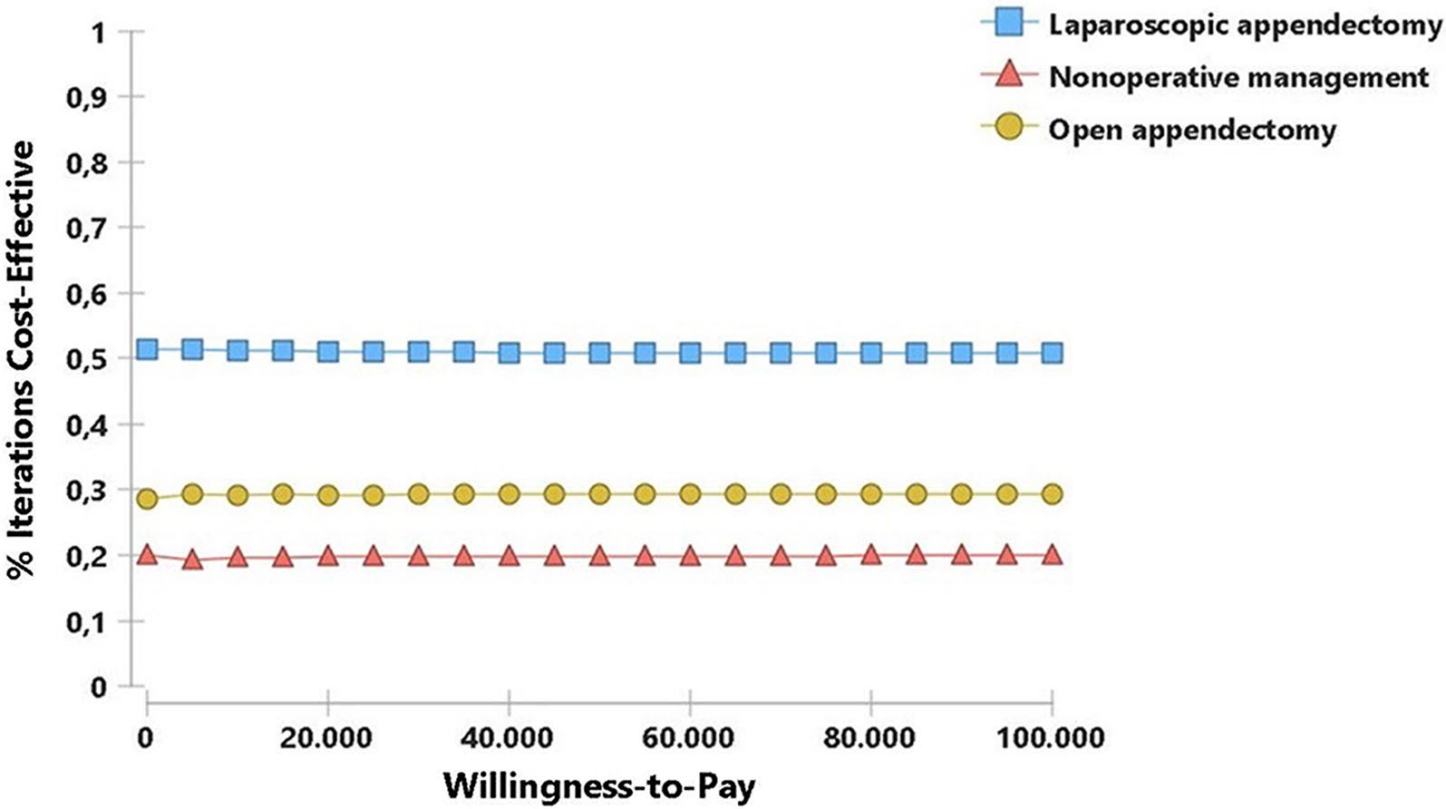
Cost effectiveness acceptability curve

The higher efficiency of LA was consistent when incorporating uncertainty into the model. The acceptability curve in Fig. 2 plots the proportion of iterations in which each alternative had the greater NMB for different willingness-to-pay. This graphic shows that LA has a 52% probability of generating the highest NMB versus its counterparts, followed by NOM (30%) and OA (18%). The choice of LA is not modified by greater or lesser willingness to pay or resource availability.

**Fig. 3:**
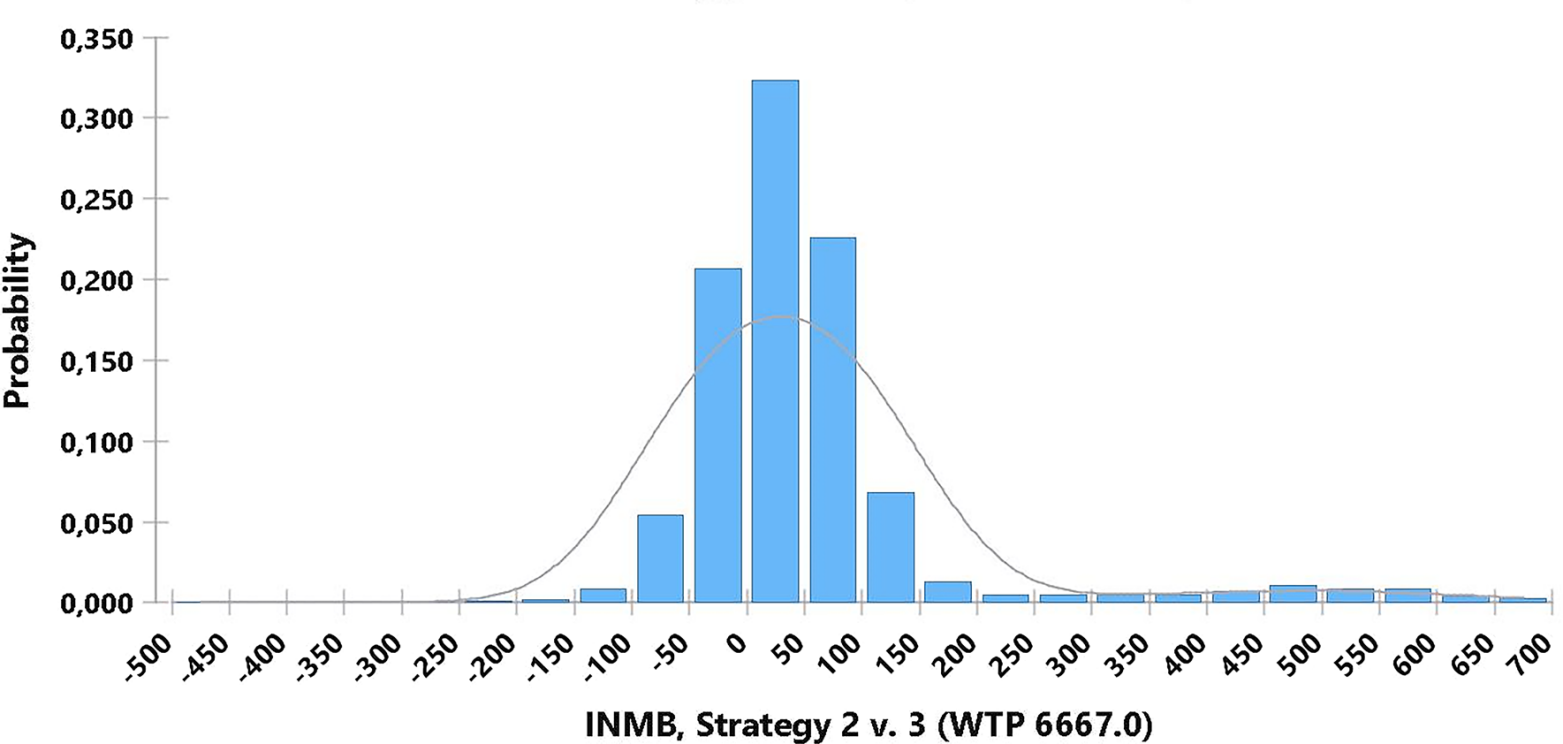
Distribution of incremental net monetary benefit laparoscopy vs medical treatment

Figure 3 sets out the incremental NMB between LA and NOM (i.e., the subtraction of the NMB of the two alternatives) for a willingness to pay 1 GDP per capita. This graph shows a probability of 0.69 that laparoscopy generates more significant benefit than medical management. The mean value of that incremental NMB would be $93.7 per patient. That mean value increases to $180 if the willingness to pay increases to 3 GDP per capita. See Addditional file 5: Figure S21.

Simulation results with 100,000 trials produced costs of $36,220,164 (± 3,422,719) with LA, $38,374,539 (± 4,030,340) with OA, and $38,194,731 (± 4,451,123) with NOM. Thus, performing OA and NOM instead of LA results in over expenditures on average of $2,154,375 and 1,974,567 per 100,000 patients. Histograms are in Addditional file 5: Figures S1–S8.

## Discussion

Our study results show that LA has a high probability of producing higher NMB than OA and NOM in patients with uncomplicated AA with a willingness to pay less than 1 GDP capita.

The lower cost of LA could explain this higher benefit. Previous studies have shown that LA might have lower costs than the open approach. These studies suggest that the reduced cost is explained by the shorter length of hospital stay, quicker postoperative recovery, and the lower probability of postoperative complications [5, 24, 51–53]. In this sense, the results of our model are consistent with those reported in the literature.

Likewise, our model revealed that the costs of LA are lower than NOM. This result differs from other published economic evaluations, which have demonstrated that NOM is cost-saving compared to surgical alternatives. This difference could be explained by the different time horizons of these studies. Sippola et al. followed some participants in the APPAC study for 1 year [3]. On the other hand, Wu et al. designed a decision model with a 1-year time horizon [23]. By adopting a shorter time horizon, the costs of patients who will recur after the first year would not be estimated. Salminen et al. demonstrated that the percentage of people who underwent appendectomy could increase from 27 to 40% after the first year [13]. Of these patients, almost 10% might recur with complicated appendicitis, which implies higher costs subsequently. Sceats et al*.* revealed that NOM was associated with higher abscess rates, readmission, and higher overall care costs [54]. As long as the appendix remains, the risk of recurrence is latent.

LA might represent significant savings for the health care system. Our model estimated a mean value of more than 2 million savings for every 100,000 LA performed instead of OA and more than 1 million compared to NOM. The probability of achieving these savings might be greater than 64%. This saving would represent 2.3% and 1.4% respectively of the total health expenditure for many people in Colombia [55]. From a financial perspective, LA might be the best option for the overall health care system, especially the secondary and third care level.

A meaningful reduction in the cost of the NOM could become a more efficient alternative than LA. According to our model, this objective would be attained by reducing NOM costs by 55%. In this case, the main expense was represented by the antibiotic regimen. Potentially, the use of other lower-cost antibiotic regimens might improve efficiency. Unfortunately, the lack of long-term comparative studies of antibiotic regimens is a limitation.

On the other hand, NOM provided a minimal increase in QALYs than the surgical alternatives. Our model revealed that NOM provided 0.003 and 0.0086 QALYs more than LA and OA, respectively. That marginal health benefit is equivalent to having 1 and 3.1 days in perfect health compared to LA and OA. Among the factors that could explain these differences are the lower probability of adverse events, less pain, and shorter absence from work than surgical options. In addition to a significant proportion who do not present symptoms or recurrence [42]. Another reason is the surgery could affect the quality of life for up to a month after being carried out [56].

Despite the above results, NOM of uncomplicated appendicitis might represent an efficient alternative in some situations. The first situation would be in case of lack of surgeons in some regions. In this case, the additional costs of transporting the patient to a center would result in LA not being the most efficient alternative. Also, the delay in the treatment would increase the risk of complications. The second situation is when the only option available is OA. This situation is frequent in hospitals where there is neither the technology nor the surgeons trained to perform LA. Two other possible situations would be in patients with high surgical risk or a collapse of the health system due to extreme public health situations (e.g., COVID). However, these two situations were not evaluated by our model.

Our economic evaluation has several limitations. Firstly, we considered a time horizon of 5 years only. This consideration might underestimate costs or overestimate QALYs in NOM because subsequent events are not quantified. Nevertheless, the incidence of this disease tends to decrease in the older population [57, 58]. Secondly, our evaluation did not include an analysis by the patients' subgroup. Possibly, NOM may be a more efficient alternative in patients with high surgical risk (e.g., older patients). To incorporate heterogeneity in baseline health states, we performed a patient-level simulation with different utilities. In a particular manner, these microsimulations could partially reflect worse health states due to comorbidities. Another limitation is the uncertainty of the inputs. Although the clinical data were drawn from good quality meta-analyses, the absence of utility and disutility data in our country could over-or underestimate the QALYs. Additionally, using uniform distribution to model costs does not represent the best option for this type of data. Finally, our model did not consider LA's learning curve and its impact on costs, post-surgical complications, or conversion to OA. The model also did not value capital costs (i.e., fiber optics, monitors) or some of the supplies needed to perform LA. Published studies show that these factors can increase costs and, therefore, could decrease this surgical approach's efficiency [59, 60].

Several issues remain to be clarified. The first one is about the probability of recurrence after five years and the impact on the quality of life and costs. On the other hand, it is mandatory quantifying the alternatives' efficiency in populations with high surgical risk or difficulty (e.g., obesity, pulmonary diseases). We consider it necessary for future research to obtain data on NOM in developing countries. These data include the clinical effectiveness of other cheaper antibiotic schemes, the estimated risk of recurrence in patients undergoing NOM, the risk of complicated appendicitis, and the probability of performing NOM in settings with a shortage of surgeons. Finally, it is necessary to incorporate into the new evaluations the effect on the costs of the LA learning curve's efficiency.

## Conclusions

LA is a cost-effectiveness alternative in the management of patients with uncomplicated AA. Besides, LA has a high probability of producing more significant monetary benefits than NOM and OA from the payer´s perspective in the Colombian health system.

## Supplementary Information

**Additional file 1: Table S1.** External validation of the model. This file shows the critical parameter used in the external validation.

**Additional file 2: Table S2.** Resources used. This file shows the resources, quantities, and values used to calculate the cost in the model.

**Additional file 3: Table S3.** Probabilities and utilities. This file shows the probabilities and utilities used in the model.

**Additional file 4: Table S4.** Distributions for PSA. This file shows the distributions and their parameter used in probabilistic sensitivity analysis.

**Additional file 5. **This file shows the histograms for the outputs of the model.

## Data Availability

All data generated or analyzed during this study are included in this published article.

## Abbreviations

AA: Acute appendicitis
OA: Open appendectomy
LA: Laparoscopic appendectomy
NOM: Nonoperative management
QALYs: Quality-adjusted life years
ICER: Incremental cost-effectiveness ratio
NMB: Net monetary benefit
