# Supplementary material for: Cost-effectiveness analysis of nonoperative management versus open and laparoscopic surgery for uncomplicated acute appendicitis in Colombia
Source: Cost Eff Resour Alloc. 2021 Jun 10;19:34. doi: 10.1186/s12962-021-00288-2 (PMC8194214; doi:10.1186/s12962-021-00288-2)
Supplement: Supplementary file 1 — Additional file 1: Table S1. External validation of the model. This file shows the critical parameter used in the external validation. [file 12962_2021_288_MOESM1_ESM.docx]

**Table S1. External validation of the model**

| Parameters for validation | Study | | | Model | | | References |
| --- | --- | --- | --- | --- | --- | --- | --- |
|  | Estimation | 95% CI | | Estimation | 95% CI | |  |
| Recurrence in NOM at 5 years | 39,1 | 33,1 | 45,3 | 33,9 | 33 | 34,8 | (14) |
| Complicated appendicitis within 5 years^1^ | 2,4 | -0,009 | 0,056 | 3,1 | 2,6 | 3,3 |  |
| Complications open appendectomy | 20,5 | 15,3 | 26,4 | 15,4 | 14,7 | 16,1 | (63) |
| Complications laparoscopic appendectomy | 9,9 | 8,415 | 25,872 | 10,6 | 9,95 | 11,2 |  |

^1 Calculated from Reference^
