# Supplementary material for: Cost-effectiveness analysis of nonoperative management versus open and laparoscopic surgery for uncomplicated acute appendicitis in Colombia
Source: Cost Eff Resour Alloc. 2021 Jun 10;19:34. doi: 10.1186/s12962-021-00288-2 (PMC8194214; doi:10.1186/s12962-021-00288-2)
Supplement: Supplementary file 2 — Additional file 2: Table S2. Resources used. This file shows the resources, quantities, and values used to calculate the cost in the model. [file 12962_2021_288_MOESM2_ESM.docx]

**Table 2. Model parameter inputs**

**Resources**

| **Input** | **Quantity** | **Unitary value** | **Total cost** | **References** |
| --- | --- | --- | --- | --- |
| **Open appendectomy** |  |  |  |  |
| *The package includes pre-surgical evaluation by the surgeon and anesthesiologist, performance of the procedure, post-surgical controls, operating room fees, surgical supplies (e.g., gauze, compresses, disposable clothing), and post-surgical hospitalization days* | 1 | 294,37 | 294,37 | (38) |
| **Laparoscopic appendectomy** |  |  |  |  |
| *Includes pre-surgical evaluation by the surgeon and anesthesiologist, performance of the procedure, post-surgical controls, operating room fees, surgical supplies (e.g., gauze, compresses, disposable clothing), and post-surgical hospitalization days* | 1 | 294,37 | 294,37 | (38) |
| *Single disposable trocar* | 1 | 113,60 | 113,60 |  |
| Total laparoscopic appendectomy |  |  | 407,97 |  |
| **Non-operative management** |  |  |  |  |
| *Hospitalization* | 3,23 | 77,34 | 249,80 | Expert, (38,62) |
| *Antibiotics* |  |  |  |  |
| Ertapenem (1gr c/24h x 3 days) | 3 | 33,38 | 100,14 |  |
| Levofloxacine (500mg c/24h x 7 days) | 7 | 3,23 | 22,59 |  |
| Metronidazol (500mg c/8h x 7 days) | 21 | 0,22 | 4,53 |  |
| *Analgesics* |  |  |  |  |
| Paracetamol (500m c/8h) | 9,69 | 0,06 | 0,58 |  |
| Dipirona IV (1g c/8h) | 9,69 | 0,15 | 1,42 |  |
| *Diagnostic aids* |  |  |  |  |
| Cell blood count | 1,615 | 3,84 | 6,20 |  |
| C reactive protein | 1,615 | 1,81 | 2,92 |  |
| *Surgeon visits* | 3,23 | 36,52 | 117,94 |  |
| *Fluids* | 6,46 | 0,41 | 2,68 |  |
| Total non operative management |  |  | 508,79 |  |
| **Post-operative complications** |  |  |  |  |
| ***Wound infection*** |  |  |  |  |
| *Cefalexin (500 mg c/8 h x 7 days)* | 21 | 0,09 | 1,93 | (62) |
| *Paracetamol (500m c/8h x 7 days)* | 21 | 0,06 | 1,25 |  |
| *Outpatient consultation* | 2 | 2,52 | 5,03 |  |
| Total wound infection |  |  | 8,21 |  |
| ***Intra-abdominal infection*** |  |  |  |  |
| *Hospitalization (days)* | 6,6 | 77,34 | 510,43 |  |
| *Percutaneous drainage guided by ultrasound* | 1 | 33,49 | 33,49 |  |
| *Ceftriaxone (1 gr c/12 h x 7 days)* | 14 | 0,65 | 9,07 |  |
| *Metronidazole (500 mg c/8 h x 7 days)* | 21 | 0,05 | 0,97 | (62) |
| *Paracetamol (500m c/8h x 7 days)* | 21 | 0,06 | 1,25 |  |
| *Cell blood count* | 3,3 | 3,84 | 12,67 |  |
| *C reactive protein* | 0,5 | 1,81 | 0,90 |  |
| *Surgeon visits* | 6,6 | 36,52 | 241,00 |  |
| Total intra-abdominal infection |  |  | 809,79 |  |
| ***Ileus*** |  |  |  |  |
| *Hospitalization (days)* | 11,6 | 77,34 | 897,12 |  |
| *Nasogastric tube* | 1 | 3,25 | 3,25 |  |
| *Intravenous fluids* | 23,2 | 0,41 | 9,61 |  |
| *Paracetamol (500m c/8h x 7 days)* | 21 | 0,06 | 1,25 | (62) |
| *Dipirona IV (1g c/8h x 7 days)* | 21 | 0,15 | 3,07 |  |
| *Cell blood count* | 5,8 | 3,84 | 22,26 |  |
| *C reactive protein* | 0,5 | 1,81 | 0,90 |  |
| *Surgeon visits* | 11,6 | 36,52 | 423,58 |  |
| *Electrolytes test* | 5,8 | 8,13 | 47,17 |  |
| Total ileus |  |  | 1408,21 |  |
| **Complicated open appendectomy** |  |  |  |  |
| *Appendectomy* | 1 | 294,37 | 294,37 |  |
| *Hospitalization (days)* | 8,9 | 77,34 | 688,30 |  |
| *Ertapenem (1 gr daily x 10 d)* | 10 | 33,38 | 333,79 |  |
| *Intravenous fluids (saline solution)* | 17,8 | 0,41 | 7,37 | (62) |
| *Paracetamol (500mg c/8h x 8 d)* | 24 | 0,06 | 1,43 |  |
| *Dipyrone IV (1g c/8h x 8 d)* | 24 | 0,15 | 3,51 |  |
| *Cell blood count* | 4,45 | 3,84 | 17,08 |  |
| *Reactive C protein* | 4,45 | 1,81 | 8,03 |  |
| *Surgeon visits* | 8,9 | 36,52 | 324,99 |  |
| Total complicated AA |  |  | 1678,88 |  |
