# Supplementary material for: Cost-effectiveness analysis of nonoperative management versus open and laparoscopic surgery for uncomplicated acute appendicitis in Colombia
Source: Cost Eff Resour Alloc. 2021 Jun 10;19:34. doi: 10.1186/s12962-021-00288-2 (PMC8194214; doi:10.1186/s12962-021-00288-2)
Supplement: Supplementary file 3 — Additional file 3: Table S3. Probabilities and utilities. This file shows the probabilities and utilities used in the model. [file 12962_2021_288_MOESM3_ESM.docx]

**Table 3. Model parameter inputs**

**Probabilities, relative risk and utilities**

| **Input** | **Value** | **Lower value** | **Upper value** | **Reference** |
| --- | --- | --- | --- | --- |
| **Probabilities and Relative risk** |  |  |  |  |
| Probability of choice OA | 0.44 |  |  | (10) |
| Probability of appendicitis within 5 years in patients that undergo NOM | 0.36 |  |  | (12) |
| Probability of complicated appendicitis within 5 years in patients that undergo NOM | 0.096 |  |  |  |
| Probability of IAA of uncomplicated LA | 0.169 |  |  | (20) |
| Probability of IAA of complicated LA | 0.047 |  |  | (32) |
| Probability of IAA in OA in uncomplicated appendicitis | 0,13 |  |  | (32) |
| Probability of wound infection of OA in complicated appendicitis | 0.128 |  |  | (32) |
| Probability of ileus in OA | 0.019 |  |  | (47) |
| Probability of ileus in LA | 0.193 |  |  |  |
| Probability of surgery if recurrence in patients after NOM | 0.60 | 0.56 | 0.64 | (10) |
| Probability of OA as initial management | 0.39 | 0.13 | 0.75 |  |
| Probability of postoperative complications of OA in complicated appendicitis | 0.22 |  |  | (32) |
| Probability of postoperative complications in LA | 0.096 |  |  | (48) |
| Probability of postoperative complications in LA in complicated appendicitis | 0.095 |  |  | (32) |
| Probability of postoperative complications of OA | 0.13 |  |  | (48) |
| Probability of recurrence | 0.063 |  |  | (49–51) |
| Probability of successful in NOM | 0.86 |  |  | (49) |
| Probability of wound infection of OA in uncomplicated appendicitis | 0.66 | 0.077 | 0.0855 | (16) |
| Probability of wound infection of OA in complicated appendicitis | 0.128 |  |  | (32) |
| Probability of wound infection of LA in uncomplicated appendicitis | 0.297 |  |  | (16,21,47) |
| Probability of wound infection of LA in complicated appendicitis | 0.131 |  |  | (32) |
| RR† complications of LA | 0.74 | 0.55 | 0.98 | (48) |
| RR of IAA in LA | 1.65 | 1.12 | 2.43 | (32) |
| RR of IAA in LA in complicated appendicitis | 1.02 | 0.71 | 1.47 | (32) |
| RR of ileus in LA | 0.91 |  |  | (47) |
| RR of postoperative complications in LA in complicated appendicitis | 0.43 | 0.31 | 0.59 | (32) |
| RR of wound infection in LA | 0.45 | 0.34 | 0.59 | (16,21,47) |
| RR of wound infection in LA in complicated appendicitis | 0.26 | 0.19 | 0.36 | (32) |
| **Utilities** |  |  |  |  |
| Utility of IAA | 0.64 |  |  | (41) |
| Utility of interval appendectomy | 0.91 |  |  |  |
| Utility of OA | 0.48 |  |  |  |
| Utility of LA | 0.58 |  |  |  |
| Utility of complicated appendicitis | 0.85 |  |  |  |
| Utility uncomplicated appendicitis | 0.91 |  |  |  |
| Utility ileus | 0.65 |  |  |  |
| Utility wound infection | 0.6 |  |  |  |
| Utility of NOM | 0.91 |  |  |  |
| Utility postoperative recovery | 0.7 |  |  |  |
| Utility in healthy people | 1 |  |  |  |
