# Supplementary material for: Cost-effectiveness analysis of nonoperative management versus open and laparoscopic surgery for uncomplicated acute appendicitis in Colombia
Source: Cost Eff Resour Alloc. 2021 Jun 10;19:34. doi: 10.1186/s12962-021-00288-2 (PMC8194214; doi:10.1186/s12962-021-00288-2)
Supplement: Supplementary file 4 — Additional file 4: Table S4. Distributions for PSA. This file shows the distributions and their parameter used in probabilistic sensitivity analysis. [file 12962_2021_288_MOESM4_ESM.docx]

**Table 4. Parameter distributions**

| Parameter | Type of distribution | Parameter 1 of distribution* | Parameter 2 of distribution^†^ | Parameter 3 of distribution ‡ | Reference (s) |
| --- | --- | --- | --- | --- | --- |
| Probabilities | | | | | |
| Postoperative complications of OA | Beta | 314 | 2040 |  | (64) |
| Any complications of OA | Dirichlet | 8.5 | 1.2 | 1.9 | (16,21,65) |
| RR complications of LA | Uniform | 0.55 | 0.98 |  | (64) |
| Any complications of LA | Dirichlet | 3.5 | 1.7 | 1.7 | (16,21,65) |
| NOM of appendicitis in five years | Beta | 93 | 163 |  | (14) |
| Choice of OA | Beta | 1809 | 2288 |  | (66) |
| Complicated appendicitis in five years | Beta | 9 | 84 |  | (14) |
| Operative management in complicated appendicitis | Beta | 239 | 192 |  | (45–47)  (45–47) |
| OA in complicated appendicitis | Beta | 64 | 97 |  |  |
| IAA after OA for complicated appendicitis | Beta | 135 | 2799 |  | (36) |
| RR of IAA after LA for complicated appendicitis | Uniform | 0.71 | 1.47 |  |  |
| Postoperative complications of OA for complicated appendicitis | Beta | 671 | 2285 |  |  |
| RR of postoperative complications LA for complicated appendicitis | Uniform | 0.43 | 0.59 |  |  |
| Recurrence after NOM | Beta | 6 | 88 |  | (45–47) |
| Success of NOM | Beta | 31 | 5 |  | (47) |
| Wound infection after OA for complicated appendicitis | Beta | 354 | 2411 |  | (36) |
| RR of wound infection after LA for complicated appendicitis | Uniform | 0.19 | 0.36 |  |  |
| RR of ileus after LA | Uniform | 0.57 | 1.47 |  | (67) |
| RR of IAA after LA | Uniform | 0.84 | 2.1 |  | (21) |
| Time | | | | | |
| AA (years) | Poisson | 0.0027 |  |  | (14) |
| Length of hospital stay of OA (days) | Poisson | 4.4 |  |  | (21) |
| Postoperative recuperation of OA (days) | Poisson | 16.14 |  |  | (19) |
| Surgical wound infection (years) | Poisson | 0.0191 |  |  | (68) |
| Cefalexin | Poisson | 7 |  |  |  |
| Length of hospital stay of IAA (years) | Poisson | 0.0180 |  |  | (65) |
| Length of hospital stay of LA (days) | Poisson | 3.21 |  |  | (21) |
| Length of hospital stay of OA (years) | Poisson | 0.0120 |  |  |  |
| Postoperative recuperation of OA (years) | Poisson | 0.0442 |  |  | (19) |
| Surgical wound infection (days) | Poisson | 7 |  |  | (68) |
| Length of hospital stay of IAA (days) | Poisson | 6.6 |  |  | (65) |
| Length of hospital stay of LA (years) | Poisson | 0.0087 |  |  | (21) |
| Postoperative recuperation of LA (years) | Poisson | 0.0306 |  |  | (19) |
| Length of hospital stay of OA for complicated appendicitis | Poisson | 8.9 |  |  | (36) |
| Ertapenem for complicated appendicitis | Poisson | 8.9 |  |  | (12) |
| Dipyrone for complicates appendicitis | Poisson | 26.7 |  |  | (19) |
| Length of hospital stay for ileus | Poisson | 11.6 |  |  | (15) |
| Length of hospital stay for NOM of uncomplicated appendicitis | Poisson | 3.2 |  |  | (1) |
| Time to interval appendectomy (years) | Poisson | 0.1150 |  |  | (46) |
| Ileus after complicated appendicitis (years) | Poisson | 0.0317 |  |  | (15) |
| NOM of appendicitis (years) | Poisson | 0.0273 |  |  | (1) |
| Length of hospital stay after LA for complicated appendicitis | Poisson | 6.34 |  |  | (36) |
| Utilities | | | | | |
| Healthy | Beta | 0.812 | 0.188 |  | (69) |
| Disutilities | | | | | |
| No complicated appendicitis | Beta | 0.089 | 0.911 |  | (69) |
| Postoperative of OA | Beta | 0.52 | 0.48 |  |  |
| Recuperation of appendectomy | Beta | 0.3 | 0.7 |  |  |
| Surgical wound infection | Beta | 0.4 | 0.6 |  |  |
| Intra-abdominal abscess (IAA) | Beta | 0.36 | 0.64 |  |  |
| Ileus | Beta | 0.35 | 0.65 |  |  |
| Postoperative of LA | Beta | 0.42 | 0.58 |  |  |
| Interval appendectomy | Beta | 0.09 | `0.91 |  |  |
| Complicated appendicitis | Beta | 0.15 | 0.85 |  |  |
| NOM for non-complicated appendicitis | Beta | 0.089 | 0.911 |  |  |
| Resources | | | | | |
| Acetaminophen (tablets) | Poisson | 7 |  |  | Expert |
| Medical assessment | Poisson | 7 |  |  |  |
| Percutaneous puncture | Poisson | 1 |  |  |  |
| Ceftriaxone (blisters) | Poisson | 14 |  |  | (6) |
| Metronidazole (tablets) | Poisson | 21 |  |  |  |
| Cell blood count | Poisson | 3.3 |  |  |  |
| PCR | Poisson | 0.5 |  |  |  |
| Surgeon visits for IAA | Poisson | 6.6 |  |  | Expert |
| Fluids for complicated appendicitis | Poisson | 17.8 |  |  |  |
| Cell blood count for complicated appendicitis | Poisson | 4.5 |  |  | (6) |
| PCR for complicated appendicitis | Poisson | 4.5 |  |  |  |
| Surgeon visits for complicated appendicitis | Poisson | 8.9 |  |  | Expert |
| Fluids por ileus | Poisson | 23.2 |  |  |  |
| Dipyrone for complicated ileus | Poisson | 34.8 |  |  |  |
| Cell blood count for ileus | Poisson | 5.8 |  |  |  |
| PCR for ileus | Poisson | 0.5 |  |  |  |
| Surgeon visits for ileus | Poisson | 11.6 |  |  |  |
| Electrolytes for ileus | Poisson | 5.8 |  |  |  |
| Dipyrone for NOM of non-complicated appendicitis | Poisson | 9.7 |  |  |  |
| Cell blood count for NOM of non-complicated appendicitis | Poisson | 1.7 |  |  |  |
| PCR for NOM of non-complicated appendicitis | Poisson | 1.7 |  |  |  |
| Surgeon visits for NOM of non-complicated appendicitis | Poisson | 3.23 |  |  |  |
| Fluids for NOM of non-complicated appendicitis | Poisson | 6.5 |  |  |  |
| Costs | | | | | |
| Cephalexin | Uniform | 0.0920 | 0.3810 |  | (70) |
| Acetaminophen | Uniform | 0.0595 | 0.0933 |  |  |
| Medical assessment | Uniform | 2.5172 | 5.0345 |  |  |
| Ceftriaxone | Uniform | 0.6480 | 3.8479 |  |  |
| Metronidazole | Uniform | 0.0462 | 0.2156 |  |  |
| Ertapenem | Uniform | 19.4307 | 33.3792 |  |  |
| Fluids | Uniform | 0.4140 | 0.7314 |  |  |
| Dipyrone | Uniform | 0.1460 | 0.5014 |  |  |

* Parameter 1 are: Alpha (Beta distribution), List of alphas (Dirichlet distribution), Lambda (Poisson distribution), Lower value (Uniform distribution)

† Parameter 2 are: Beta (Beta distribution), High value (Uniform distribution)

‡ Parameter 3 are: Integer/Real (Uniform and Beta distribution)
