# Supplementary material for: Cost-effectiveness analysis of nonoperative management versus open and laparoscopic surgery for uncomplicated acute appendicitis in Colombia
Source: Cost Eff Resour Alloc. 2021 Jun 10;19:34. doi: 10.1186/s12962-021-00288-2 (PMC8194214; doi:10.1186/s12962-021-00288-2)
Supplement: Supplementary file 5 — Additional file 5. This file shows the histograms for the outputs of the model. [file 12962_2021_288_MOESM5_ESM.pdf]

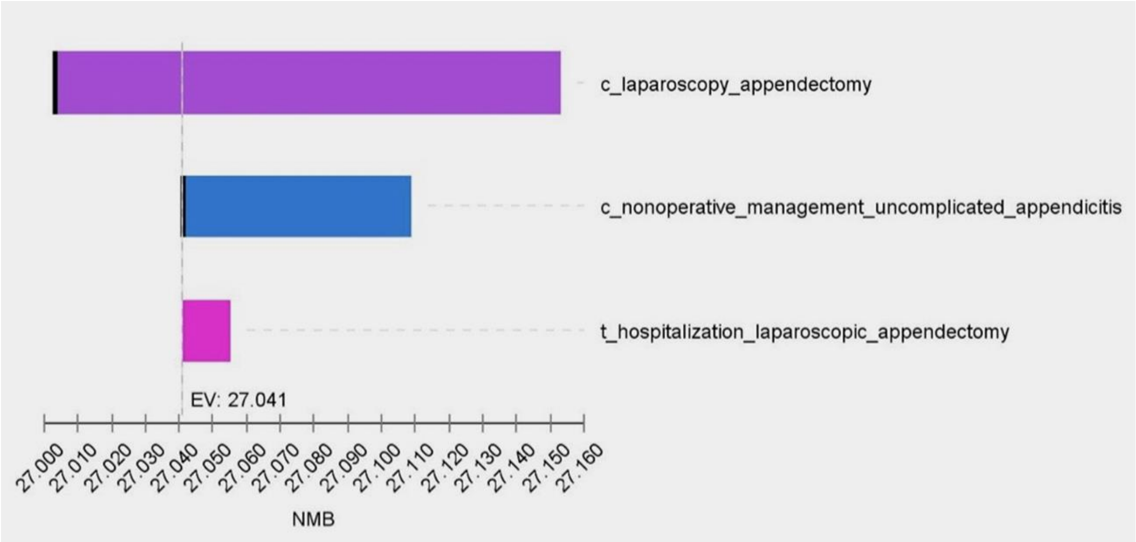

S1- TORNADO GRAPH

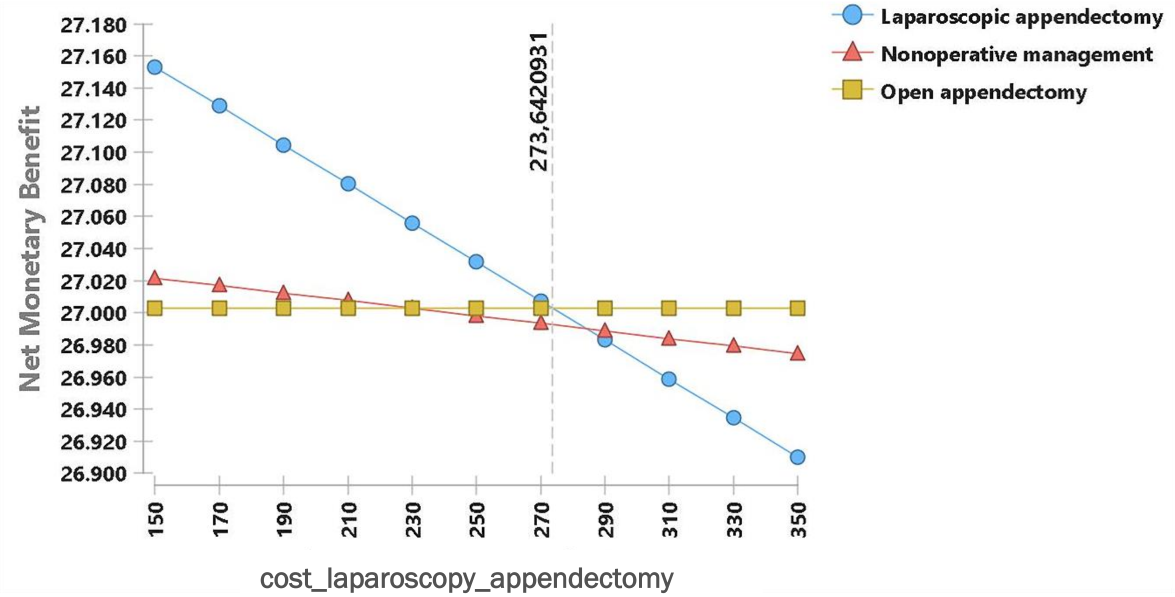

S2- THRESHOLD ANALYSIS (WTP=6.667) LAPAROSCOPIC APPENDECTOMY

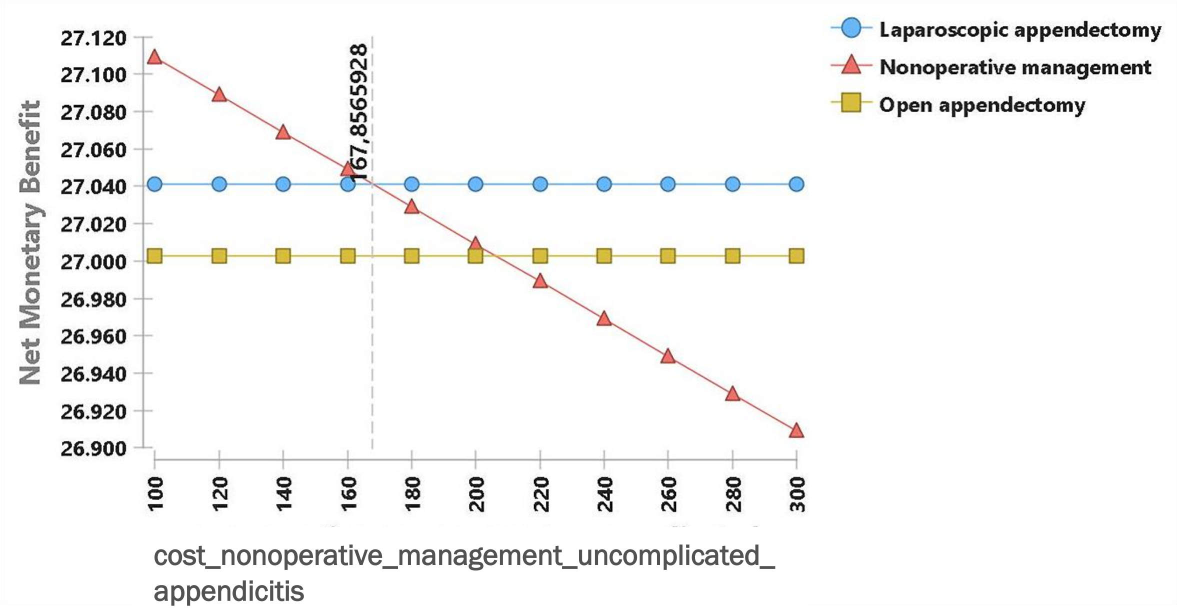

S3- THRESHOLD ANALYSIS (WTP=6.667) NONOPERATIVE MANAGEMENT

UNDISCOUNTED

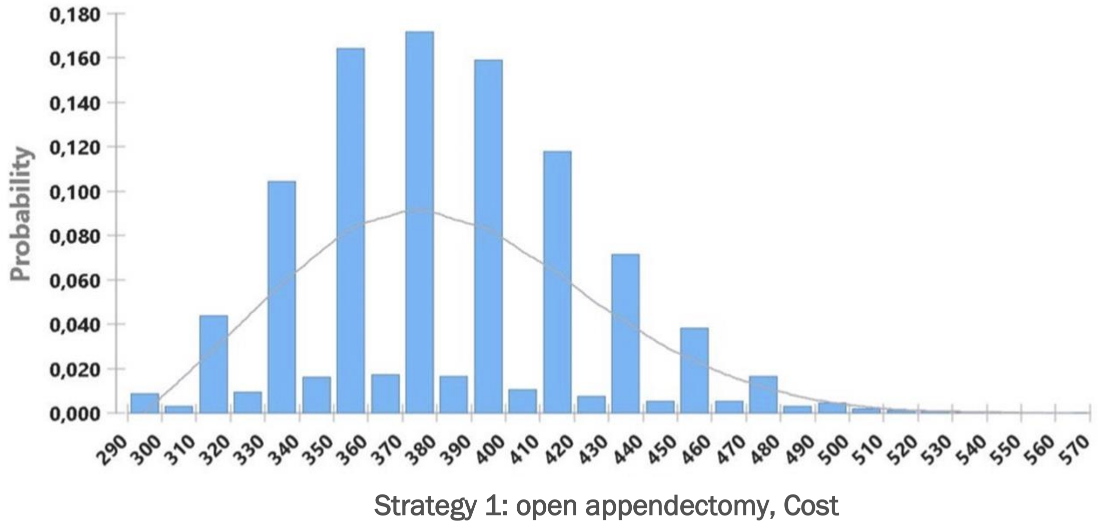

S4 – DISTRIBUTION OF COST OF OPEN APPENDECTOMY

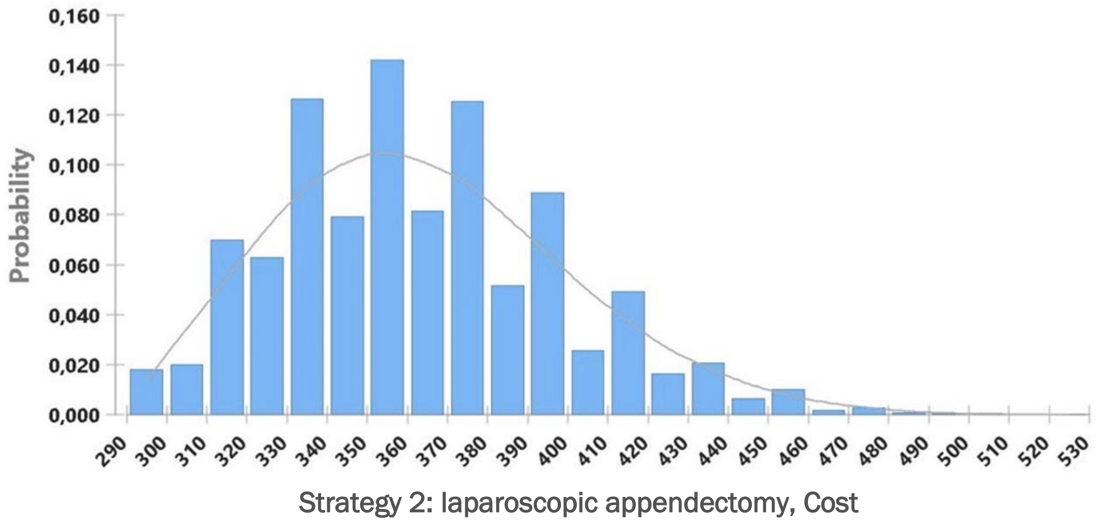

S5- DISTRIBUTION OF COST LAPAROSCOPIC APPENDECTOMY

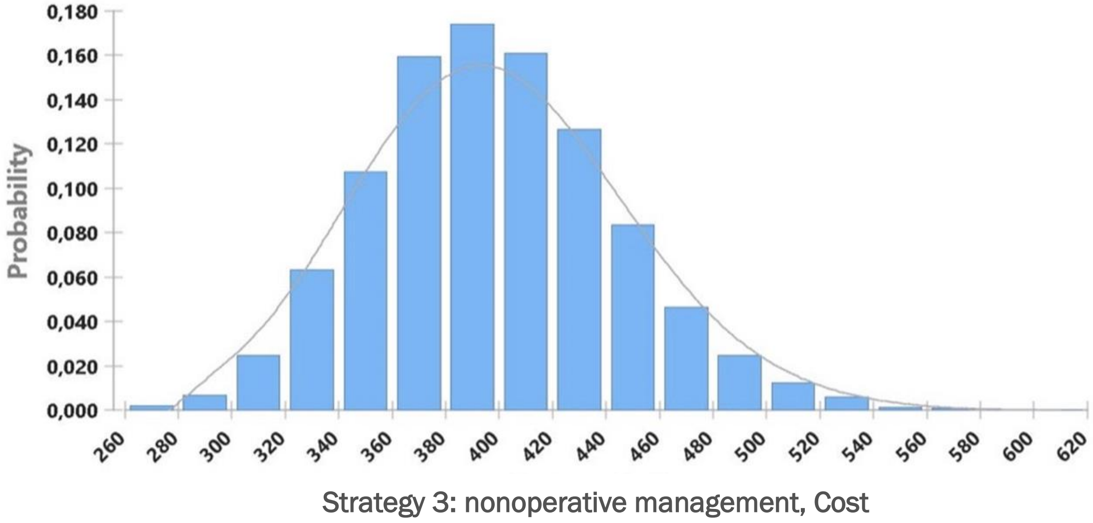

S6- DISTRIBUTION OF COST NONOPERATIVE MANAGEMENT

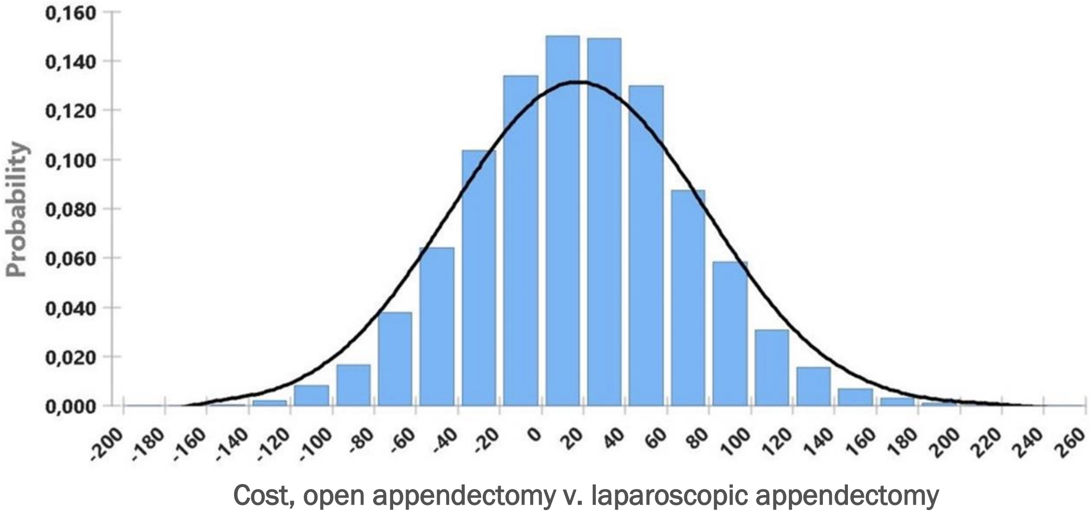

S7- DISTRIBUTION OF INCREMENTAL COST BETWEEN OPEN vs LAPAROSCOPIC APPENDECTOMY

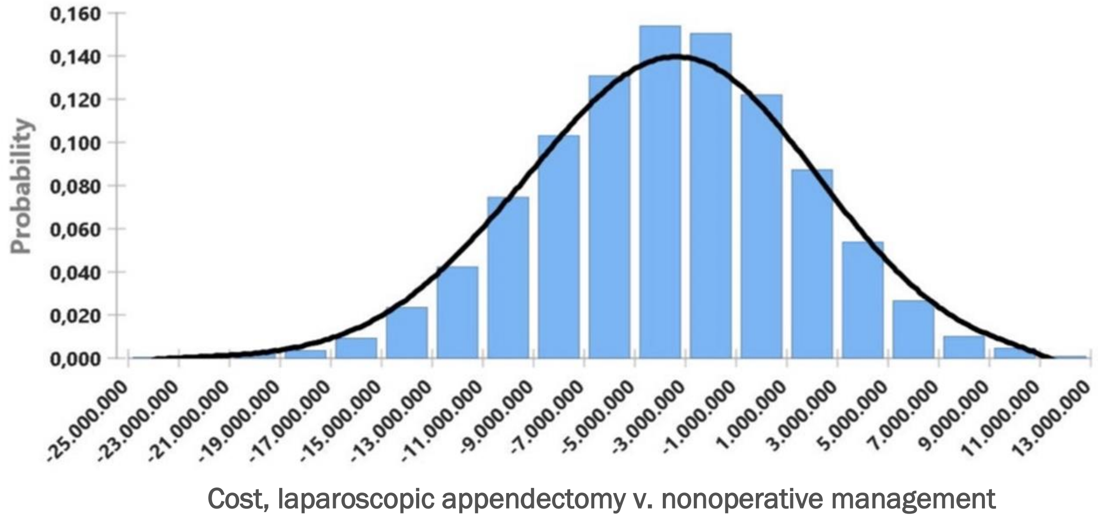

S8- DISTRIBUTION OF INCREMENTAL COST BETWEEN LAPAROSCOPIC vs NONOPERATIVE MANAGEMENT

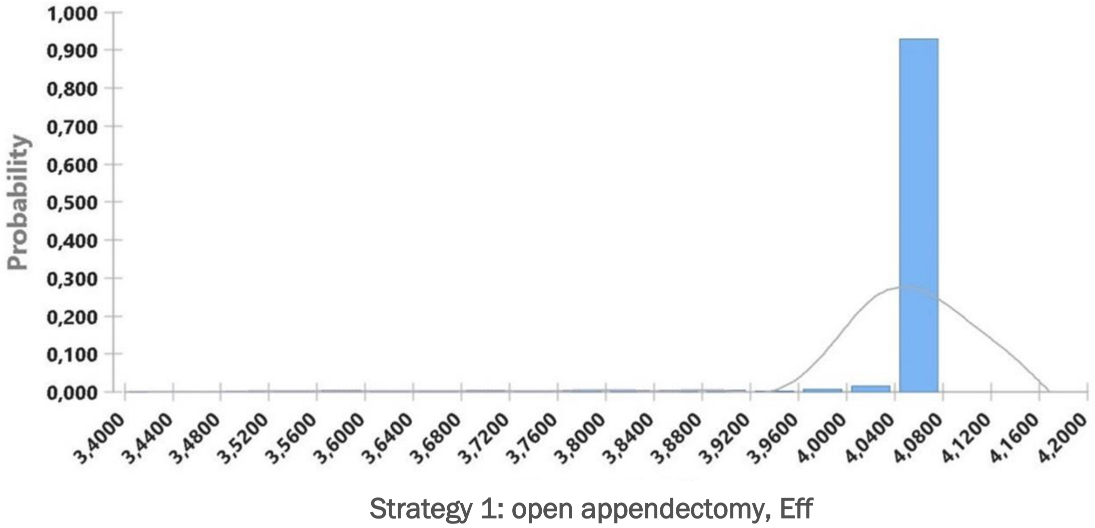

S9- DISTRIBUTION OF QALYs OPEN APPENDECTOMY

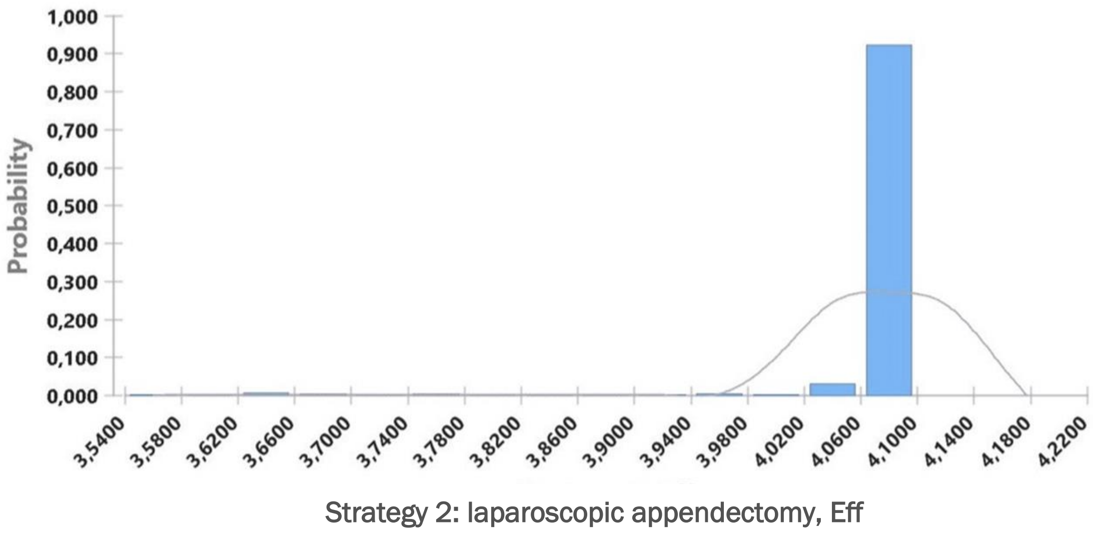

S10- DISTRIBUTION OF QALYs LAPAROSCOPIC APPENDECTOMY

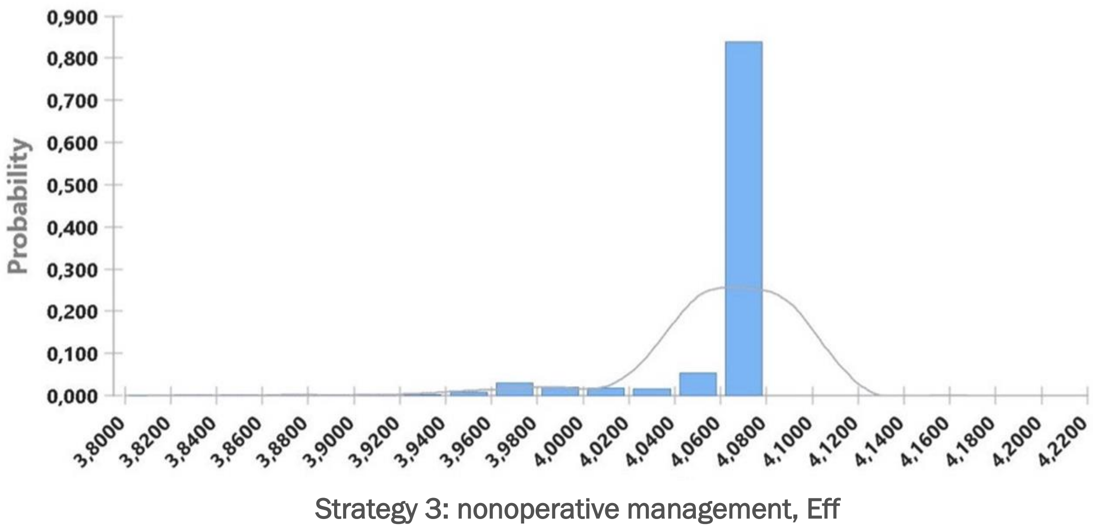

S11- DISTRIBUTION OF QALYs NONOPERATIVE MANAGEMENT

DISCOUNTED

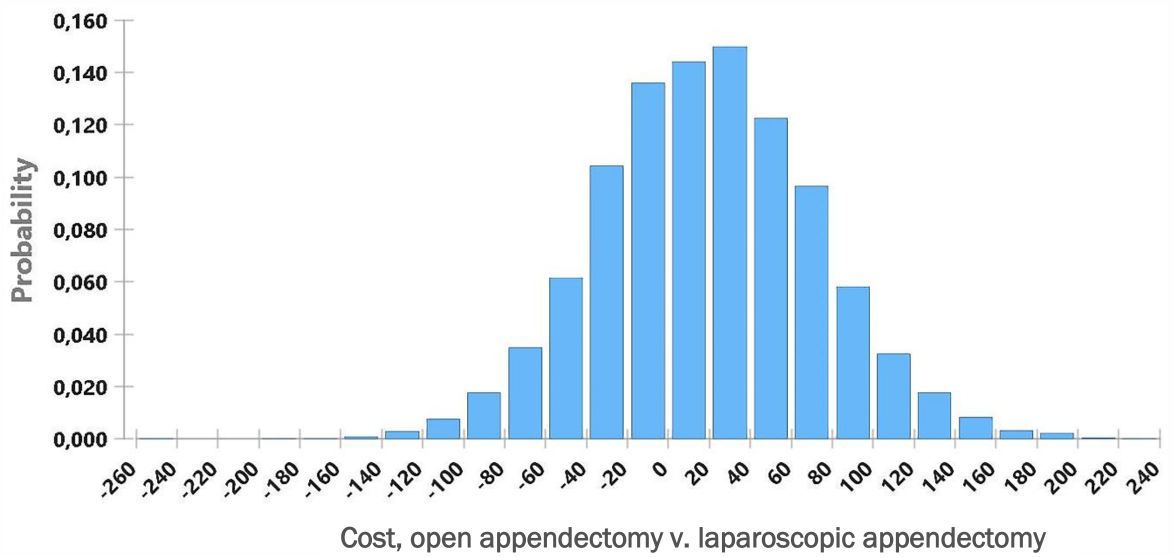

S12- DISTRIBUTION OF INCREMENTAL COST BETWEEN OPEN vs LAPAROSCOPIC APPENDECTOMY

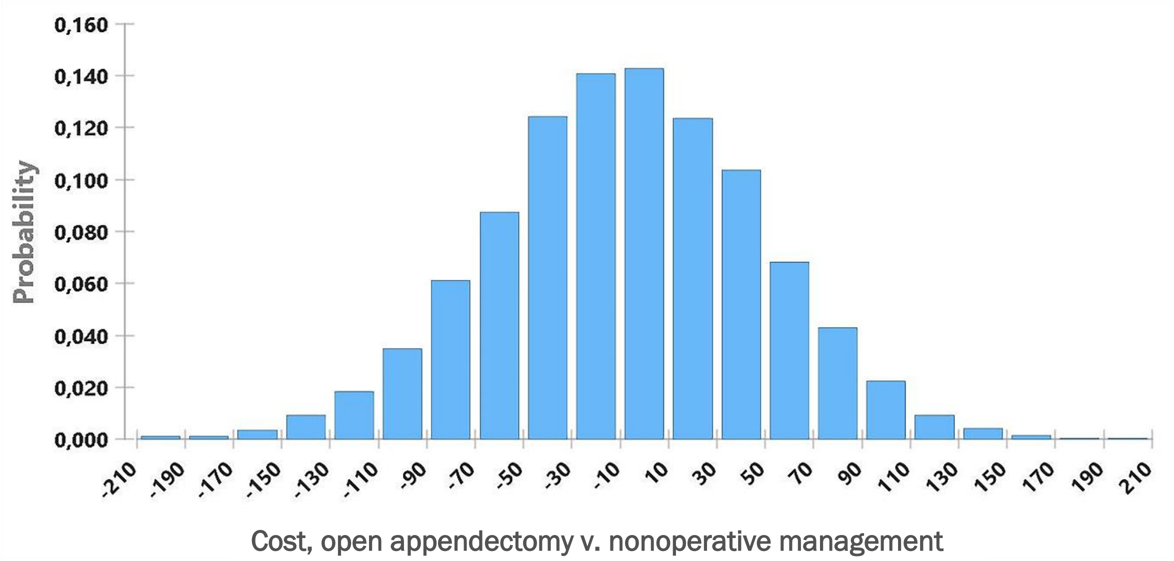

S13- DISTRIBUTION OF INCREMENTAL COST BETWEEN OPEN APPENDECTOMY vs NONOPERATIVE MANAGEMENT

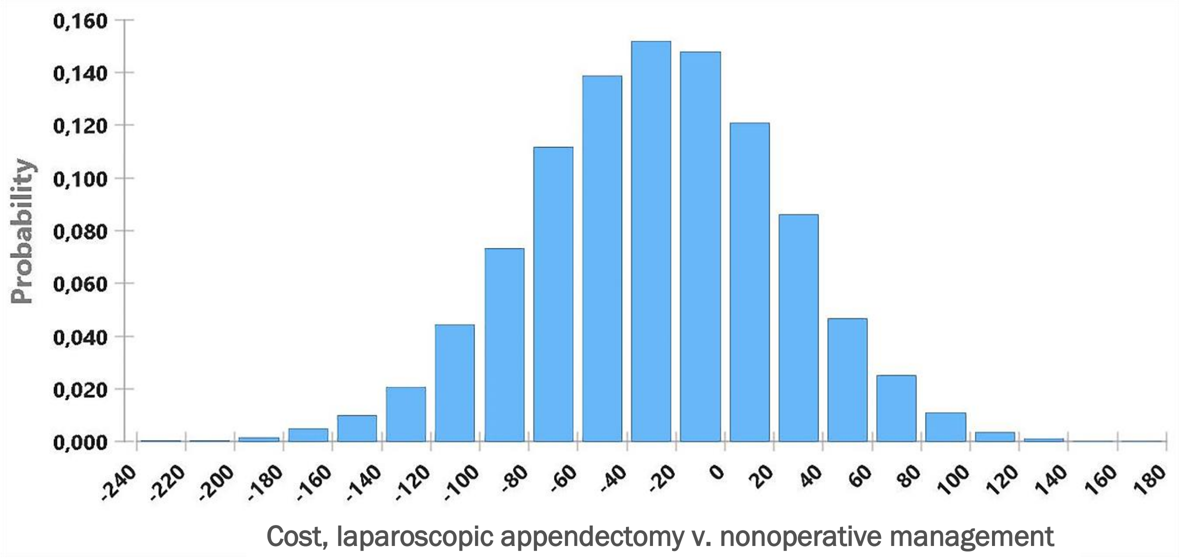

S14- DISTRIBUTION OF INCREMENTAL COST BETWEEN LAPAROSCOPIC APPENDECTOMY vs NONOPERATIVE MANAGEMENT

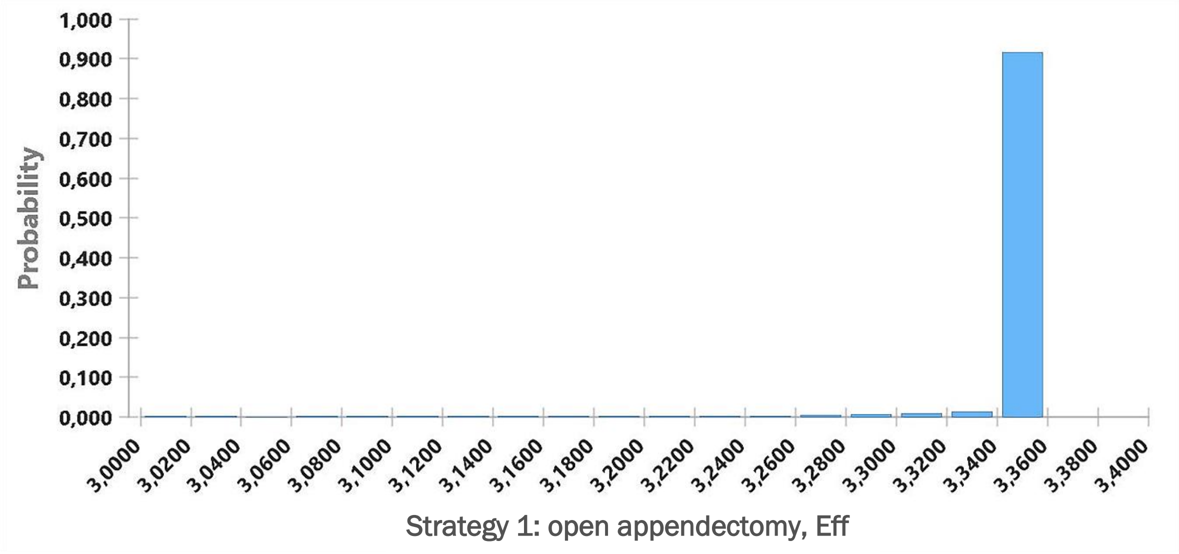

S15- DISTRIBUTION OF QALYs OPEN APPENDECTOMY

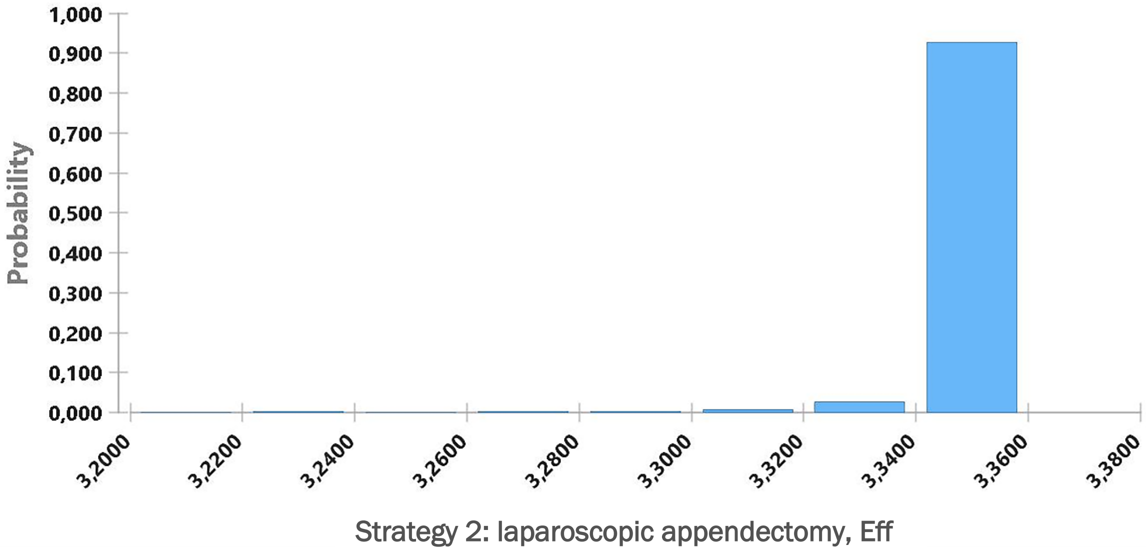

S16- DISTRIBUTION OF QALYs LAPAROSCOPIC APPENDECTOMY

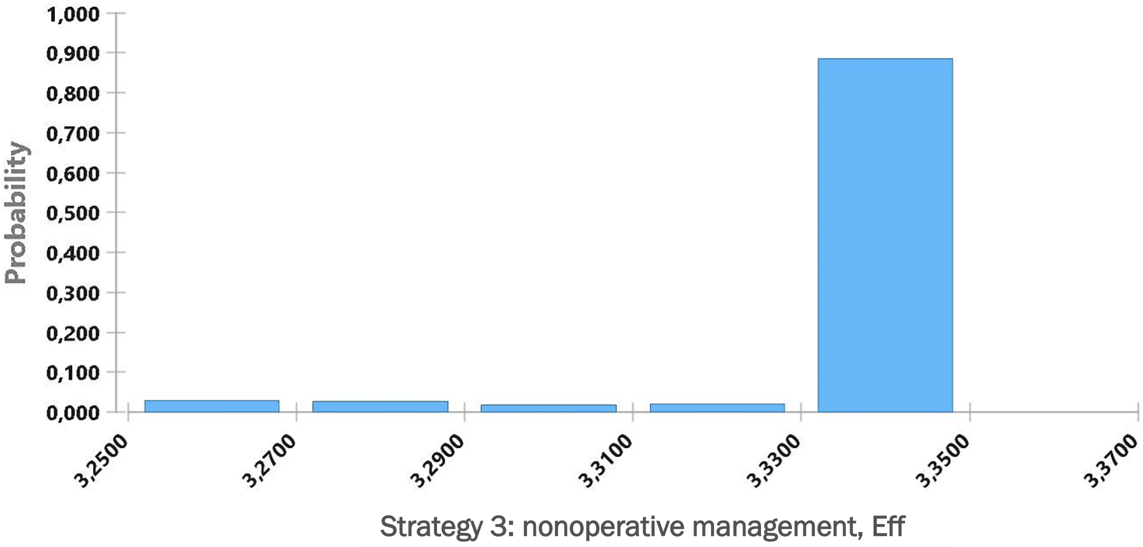

S17- DISTRIBUTION OF QALYs NONOPERATIVE MANAGEMENT

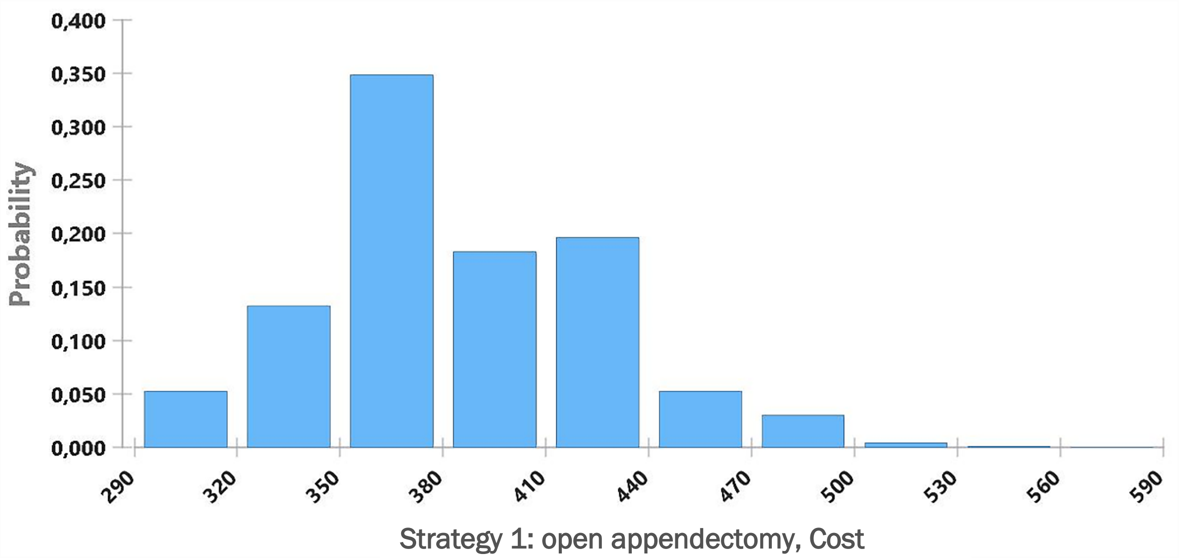

S18- DISTRIBUTION OF COST OF OPEN APPENDECTOMY

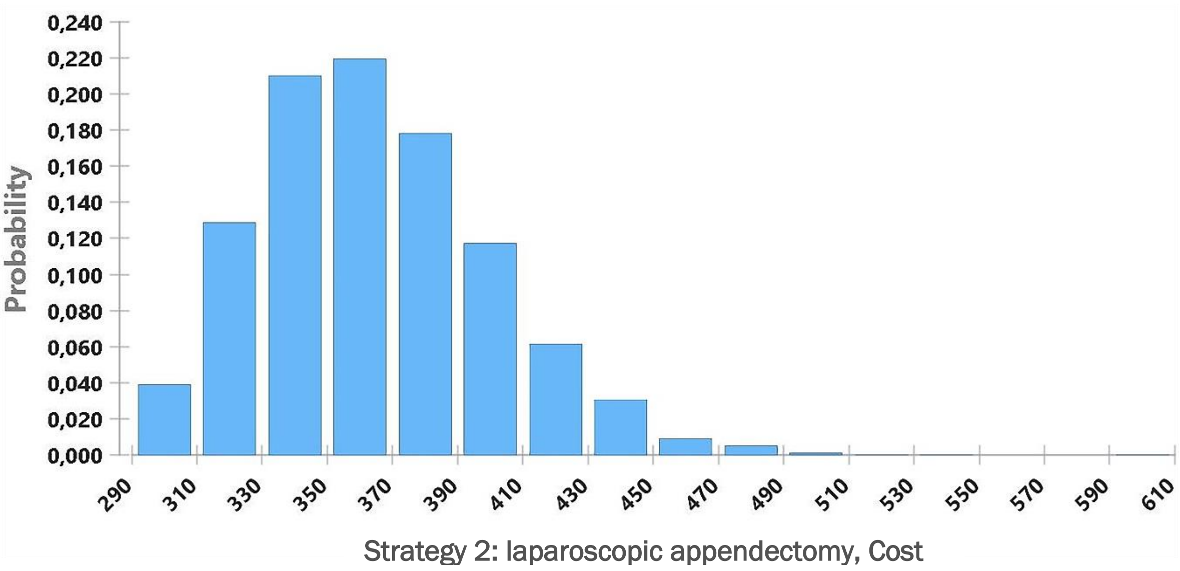

S19- DISTRIBUTION OF QALYs LAPAROSCOPIC APPENDECTOMY

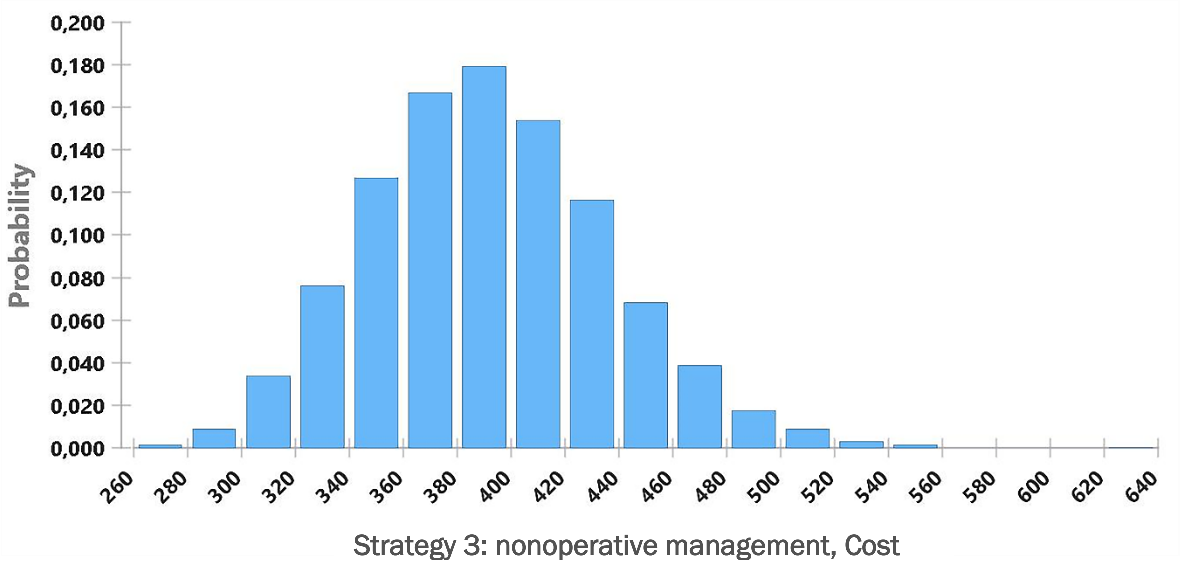

S20- DISTRIBUTION OF QALYs NONOPERATIVE MANAGEMENT

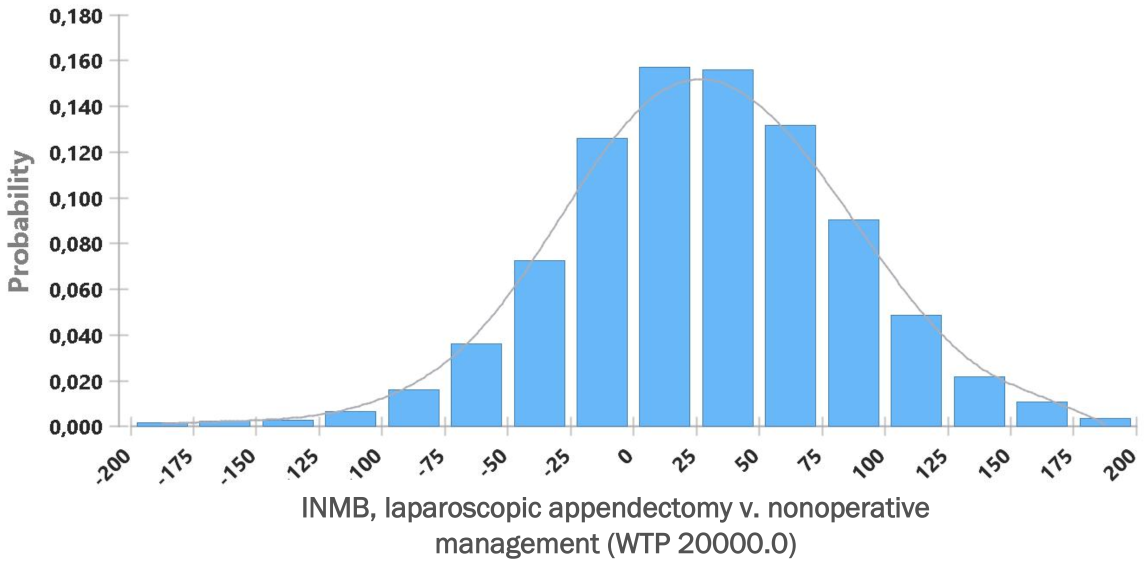

S21- DISTRIBUTION OF INCREMENTAL NET MONETARY BENEFIT BETWEEN LAPAROSCOPIC APPENDECTOMY vs NONOPERATIVE MANAGEMENT
